# Hemorrhagic shock from a gastrointestinal stromal tumor of the small intestine: an unusual case report

**DOI:** 10.1093/omcr/omag072

**Published:** 2026-06-08

**Authors:** Mustapha Dahiri, Mohammed Amine Lkousse, Hicham El Boté

**Affiliations:** Phaculty of Medicine and Pharmacy, Mghila, Béni Mellal 23000, Morocco; Phaculty of Medicine and Pharmacy, Mghila, Béni Mellal 23000, Morocco; Phaculty of Medicine and Pharmacy, Mghila, Béni Mellal 23000, Morocco

**Keywords:** Hemorrhagic shock, GIST, small intestine, emergency, surgery

## Abstract

Gastrointestinal stromal tumors (GISTs) are rare, typically sporadic connective tissue tumors, most commonly located in the stomach or small intestine. We report an unusual case of a 59-year-old female who underwent emergency surgery for hemorrhagic shock caused by a small intestinal GIST, resulting in massive hemoperitoneum. GISTs often remain asymptomatic for a long time, only becoming apparent when they enlarge or cause complications. Very rarely, these tumors present as hemoperitoneum. This case highlights that in patients with hemorrhagic shock, massive hemoperitoneum, and an abdominal mass on imaging, a bleeding GIST should be considered, despite its rarity.

## Introduction

Gastrointestinal stromal tumors (GISTs) are the most common mesenchymal tumors of the digestive tract. They can arise in any segment of the gastrointestinal tract and originate from the interstitial cells of Cajal [1, 2].

GISTs often remain asymptomatic for a long time, with clinical manifestations usually resulting from complications or a large tumor mass. Hemorrhagic shock due to massive hemoperitoneum is a rare complication [3, 4].

## Case presentation

A 59-year-old woman with well-controlled type 2 diabetes and hypertension, with no history of smoking or alcohol consumption, presented with diffuse abdominal pain. She had experienced these symptoms intermittently for eight months, without rectal bleeding, melena, or bowel habit changes, and had not sought medical attention.

**Figure 1:**
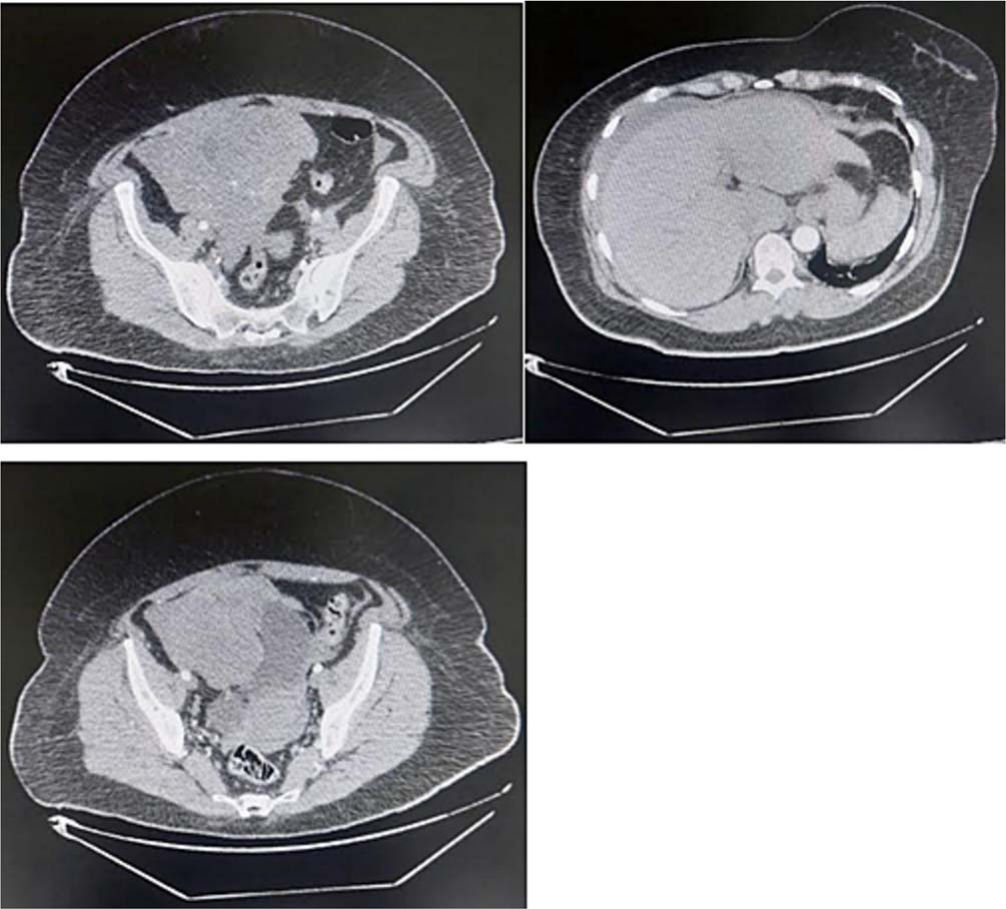
CT Scan showing the tumour mass with massive peritoneal effusion in our patient.

She was admitted to the emergency department with diffuse abdominal pain and hemorrhagic shock. CT imaging revealed a large hemoperitoneum and an abdominopelvic mass measuring 12.5 × 10.5 × 9.2 cm (Fig. 1).

**Figure 2:**
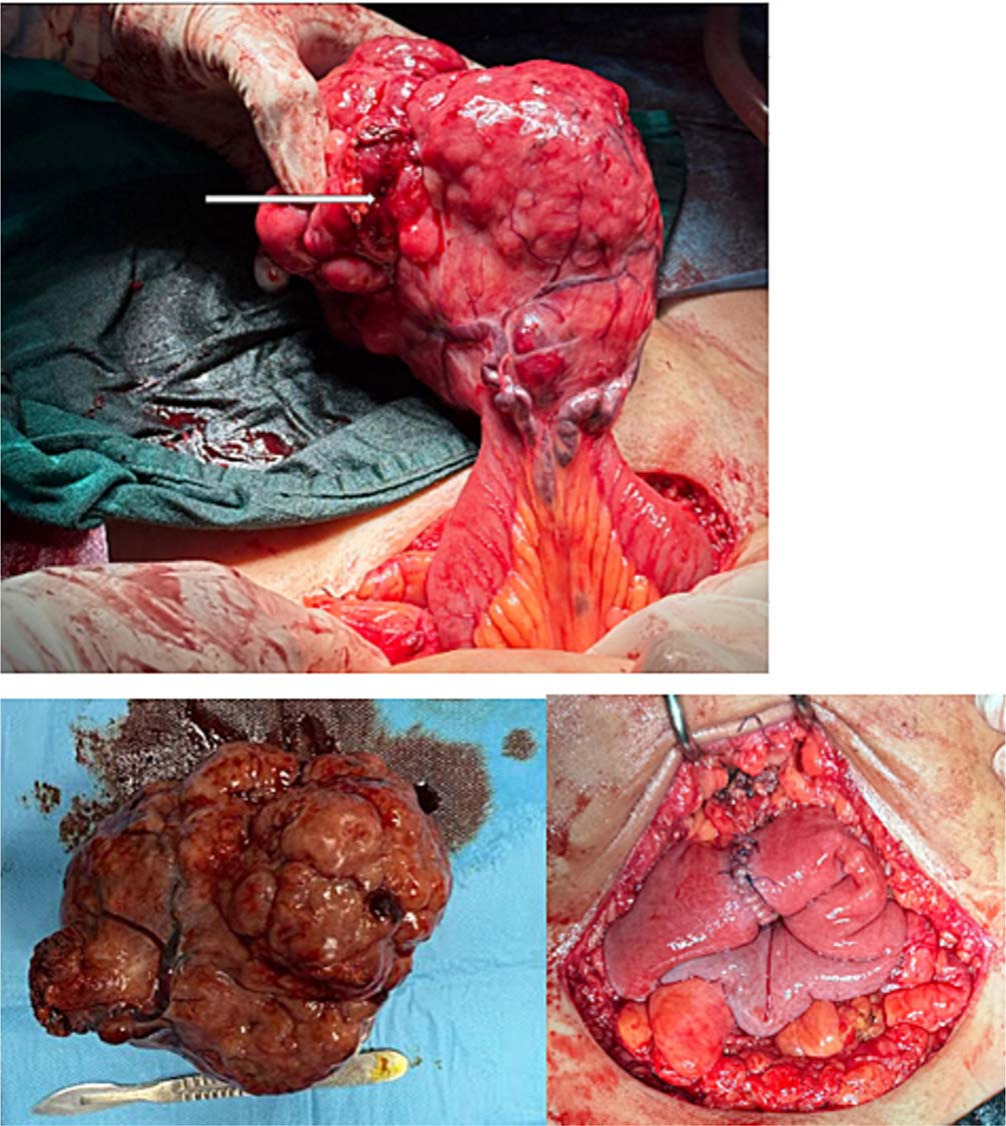
Intraoperative view of the tumour mass showing the bleeding site (arrow), the resected specimen, and the end-to-end anastomosis in our patient.

The patient was transfused and stabilized prior to laparotomy, which revealed a massive hemorrhagic peritoneal effusion and an exoluminal ileal mass bleeding from a neovessel. No secondary lesions were identified during the rest of the exploration. After aspiration of the hematic effusion, a segmental bowel resection was performed with macroscopically clear margins and an end-to-end anastomosis (Fig. 2).

The postoperative course was uneventful. Histopathology confirmed a GIST measuring 12.5 × 10.5 × 9.2 cm, with R0 resection. According to the Miettinen classification, the tumor carried a high risk of recurrence.

A completion thoracic CT scan showed no additional disease. Adjuvant imatinib therapy for five years was recommended during the multidisciplinary team meeting, along with annual imaging follow-up.

## Discussion

GISTs are rare, typically sporadic connective tissue tumors, most commonly located in the stomach or small intestine [1, 2]. Although they are the most common mesenchymal tumors of the digestive tract, GISTs account for only about 1% of all gastrointestinal tumors [5].

They originate from the interstitial cells of Cajal or their precursors and typically express KIT+ (95% of cases) and DOG-1+ (95% of cases). Oncogenic mutations in the KIT or platelet-derived growth factor receptor alpha (PDGFRA) genes, which encode receptor tyrosine kinases, are found in approximately 85% of adult GISTs [2].

About 60% of GISTs are located in the stomach, 30% in the small intestine, and around 5% in the colon or rectum [5]. GISTs may remain asymptomatic for a long period, with incidental detection in roughly 20% of cases during endoscopy, imaging, or surgery [3].

Clinical presentation varies with tumor location and may include gastrointestinal bleeding [4], non-specific abdominal pain, a palpable mass, bowel obstruction especially in small intestine tumors or, rarely, hemoperitoneum [3, 4].

Investigations depend on tumor size and location. For tumors smaller than 5 cm in the stomach or colorectum, diagnosis is typically made via endoscopy and endoscopic ultrasound. For larger masses, abdominal CT is the gold standard [6]. On imaging, the tumor usually appears as a rounded or oval mass with well-defined borders, often growing exophytically. Tumor diameter is an important prognostic factor [7].

Surgical management aims to avoid intraoperative tumor perforation, which significantly increases the risk of recurrence through peritoneal dissemination [8]. For small bowel GISTs, segmental resection with macroscopically clear margins and without lymph node dissection is recommended [1]. Surgery is essential in cases of hemoperitoneum and hemodynamic instability, as in our patient.

Assessing the risk of recurrence is crucial for determining the need for adjuvant therapy with imatinib and for planning follow-up [1, 2]. Imatinib is indicated for GISTs at high risk of recurrence after surgical resection. It inhibits the tyrosine kinase activity of the c-KIT protein, blocking signaling pathways that promote tumor cell growth and survival. This mechanism is particularly effective in GISTs with activating c-KIT mutations [1].

In our patient, the risk of recurrence was classified as high according to the Miettinen classification due to a tumor larger than 10 cm (12.5 cm) and its non-gastric location (small intestine).
